# Dehydrocostus Lactone Attenuates Methicillin-Resistant *Staphylococcus aureus*-Induced Inflammation and Acute Lung Injury via Modulating Macrophage Polarization

**DOI:** 10.3390/ijms22189754

**Published:** 2021-09-09

**Authors:** Ya-Xian Wu, Feng-Juan Jiang, Gang Liu, Ying-Ying Wang, Zhi-Qi Gao, Si-Hao Jin, Yun-Juan Nie, Dan Chen, Jun-Liang Chen, Qing-Feng Pang

**Affiliations:** 1 Wuxi School of Medicine, Jiangnan University, Wuxi 214122, China; yaxianwu@jiangnan.edu.cn (Y.-X.W.); jiangfj2020@foxmail.com (F.-J.J.); weiruishang@foxmail.com (G.L.); wangyingying2021@foxmail.com (Y.-Y.W.); gaozhiqi277@foxmail.com (Z.-Q.G.); jinsihao123@foxmail.com (S.-H.J.); nieyunjuan@jiangnan.edu.cn (Y.-J.N.); danchen09@foxmail.com (D.C.); chenjunliang572@foxmail.com (J.-L.C.); 2 School of Food Science and Technology, Jiangnan University, Wuxi 214122, China

**Keywords:** Dehydrocostus lactone, LTA, MRSA, acute lung injury, macrophage polarization

## Abstract

Dehydrocostus lactone (DHL), a natural sesquiterpene lactone isolated from the traditional Chinese herbs *Saussurea lappa* and *Inula helenium* L., has important anti-inflammatory properties used for treating colitis, fibrosis, and Gram-negative bacteria-induced acute lung injury (ALI). However, the effects of DHL on Gram-positive bacteria-induced macrophage activation and ALI remains unclear. In this study, we found that DHL inhibited the phosphorylation of p38 MAPK, the degradation of IκBα, and the activation and nuclear translocation of NF-κB p65, but enhanced the phosphorylation of AMP-activated protein kinase (AMPK) and the expression of Nrf2 and HO-1 in lipoteichoic acid (LTA)-stimulated RAW264.7 cells and primary bone-marrow-derived macrophages (BMDMs). Given the critical role of the p38 MAPK/NF-κB and AMPK/Nrf2 signaling pathways in the balance of M1/M2 macrophage polarization and inflammation, we speculated that DHL would also have an effect on macrophage polarization. Further studies verified that DHL promoted M2 macrophage polarization and reduced M1 polarization, then resulted in a decreased inflammatory response. An in vivo study also revealed that DHL exhibited anti-inflammatory effects and ameliorated methicillin-resistant *Staphylococcus aureus* (MRSA)-induced ALI. In addition, DHL treatment significantly inhibited the p38 MAPK/NF-κB pathway and activated AMPK/Nrf2 signaling, leading to accelerated switching of macrophages from M1 to M2 in the MRSA-induced murine ALI model. Collectively, these data demonstrated that DHL can promote macrophage polarization to an anti-inflammatory M2 phenotype via interfering in p38 MAPK/NF-κB signaling, as well as activating the AMPK/Nrf2 pathway in vitro and in vivo. Our results suggested that DHL might be a novel candidate for treating inflammatory diseases caused by Gram-positive bacteria.

## 1. Introduction

Bacterial infection is the main cause of acute lung injury (ALI)/acute respiratory distress syndrome (ARDS), which is characterized by severe inflammation and can result in respiratory failure [1]. *Staphylococcus aureus* (*S. aureus*), a Gram-positive bacterium, is a leading cause of infection worldwide [2] and is the main pathogenic bacterium of nosocomial pneumonia, which is related to the development of ALI/ARDS [3]. Although antibiotics are used to treat S. aureus infections, the emergence of antibiotic-resistant species such as methicillin-resistant *S. aureus* (MRSA) has resulted in the widespread reoccurrence of pneumonia, posing a daunting challenge for public health [4]. Therefore, novel effective agents against antibiotic-resistant bacteria are urgently needed.

ALI/ARDS develops through excessive and uncontrolled inflammatory responses to direct and indirect lung injury [5]. As an indispensable component of innate immunity, macrophages play a crucial role in inflammatory responses and ALI [6]. Macrophages can be polarized into two main phenotypes: the classically activated (M1) phenotype and the alternatively activated (M2) phenotype [7]. Activation of the M1 phenotype promotes inflammation, increases the level of oxidative stress-induced products and tissue damage, and is remarkable in the acute stage of inflammation. Conversely, activation of the M2 phenotype augments the production of anti-inflammatory cytokines, which contributes to decreasing inflammation and promoting tissue repair, then resulting in the resolution of inflammation [8]. Proinflammatory M1 macrophages can be classically triggered by recognition of Gram-negative bacteria (e.g., lipopolysaccharide (LPS)) or Gram-positive bacteria (e.g., *S. aureus*). During Gram-positive bacteria-triggered inflammatory responses, toll-like receptor 2 (TLR2) recruits myeloid differentiation factor 88 (MyD88), and then activates the downstream signaling cascade, including the phosphorylation of p38 mitogen-activated protein kinase (MAPK) and the degradation of IκBα, which result in activation and nuclear translocation of nuclear factor-κB (NF-κB) [9,10]. Ultimately, TLR2-mediated signaling promotes M1 phenotype macrophages, which are characterized by elevated expression of inducible nitric oxide synthase (iNOS) and increased production of proinflammatory cytokines (e.g., TNF-α, IL-1β, and IL-6) [11]. During the development of ALI, anti-inflammatory signaling pathways are also activated, such as the adenosine monophosphate-activated protein kinase (AMPK)/nuclear factor erythroid 2-releated factor 2 (Nrf2) pathway [12]. Moreover, studies showed that AMPK/Nrf2 signaling and its downstream gene heme oxygenase-1 (HO-1) contributed to the M2 macrophage phenotype [13,14] and exerted an anti-inflammatory effect. Interestingly, as macrophages are highly plastic cells, M1 macrophages can switch into M2 phenotypes (characterized by high expression of CD163 and CD206), and vice versa, depending on the microenvironment [15]. Studies revealed that the balance of the M1/M2 phenotype may determine the fate of an organ in inflammation or injury [16]. Therefore, regulating the ratio of the M1/M2 phenotype may be a therapeutic strategy to ameliorate inflammation and ALI. However, few compounds have been authenticated as having the ability to alleviate ALI by mediating the ratio of the M1/M2 phenotype, especially in Gram-positive bacteria-induced ALI.

Dehydrocostus lactone (DHL) is a natural sesquiterpene lactone extracted from *Saussurea lappa* and *Inula helenium* L., which are commonly used in traditional herbal medicine in China [17,18,19]. Accumulating studies reveal that DHL possesses multiple activities, including anticancer, antioxidant and anti-inflammatory properties [20,21,22,23]. Previous studies reported that DHL could ameliorate LPS-induced ALI by modulating macrophage activation via inhibiting the MAPK/NF-κB signaling pathway or NLRP3 inflammasome activation [18,24,25]. However, the bioactivity of DHL in Gram-positive bacteria-induced inflammatory response is poorly understood. Whether DHL has protective effects in MRSA-induced ALI also remains unclear. Lipoteichoic acid (LTA), a major pathogenic component of the Gram-positive bacterial outer cell wall, was used in an in vitro study, as it is implicated in triggering the inflammatory response and causing tissue injury [26,27]. In the present study, we examined the potential role and the underlying mechanisms of DHL in macrophage polarization. We found that DHL promoted the polarization of macrophages from M1 phenotype to M2 phenotype, and this process was involved in the p38 MAPK/NF-κB and AMPK/Nrf2 signaling pathways. Furthermore, we found that DHL had potent anti-inflammatory activity in Gram-positive bacteria-induced inflammation, and ameliorated MRSA-induced ALI in a mouse model.

## 2. Results

### 2.1. DHL Attenuates LTA-Induced Phosphorylation of p38 MAPK and NF-κB in RAW264.7 Cells and BMDMs

**Figure 1:**
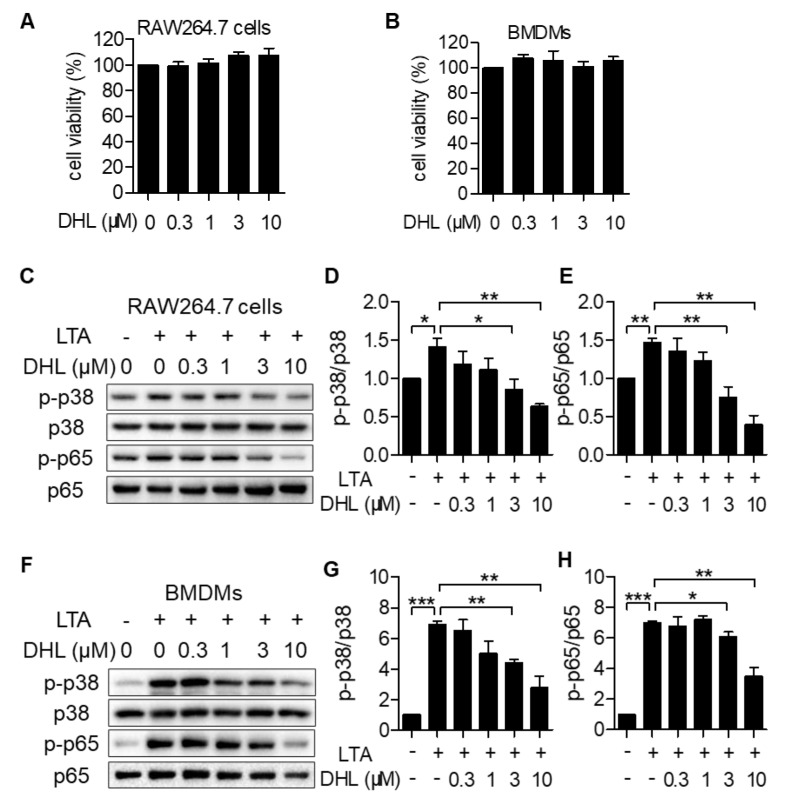

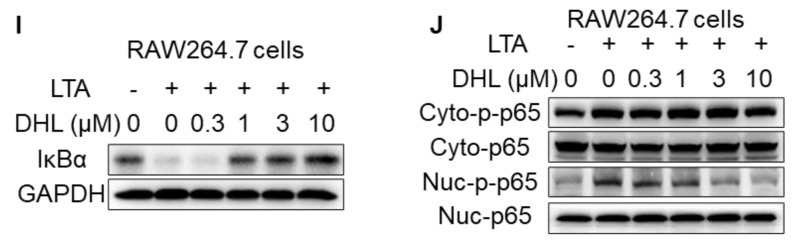
DHL reduces p38 MAPK and NF−κB phosphorylation in LTA−induced RAW264.7 cells and primary BMDMs. (**A**,**B**) CCK8 measurement of cell vitality. RAW264.7 cells (**A**) and BMDMs (**B**) were treated with different concentrations (0, 0.3, 1, 3, and 10 μM) of DHL for 24 h. RAW264.7 cells (**C**–**E**,**I**) and BMDMs (**F**–**H**) were pretreated with DHL at 0, 0.3, 1, 3, and 10 μM for 0.5 h, then stimulated with LTA (20 μg/mL) for another 0.5 h. Cell lysates were collected, and the protein expression levels of p-p38, p38, p-p65, and p65 were evaluated by Western blot. The ratios of p-p38/p38 (**D**,**G**) and p-p65/p65 (**E**,**H**) were semiquantitatively analyzed by ImageJ software. (**I**,**J**) RAW264.7 cells were pretreated with DHL at 0, 0.3, 1, 3, and 10 μM for 0.5 h, then stimulated with LTA (20 μg/mL) for another 0.5 h. Cytoplasm and nuclear fractions were extracted and subjected to immunoblot analysis using IκB antibody, p-p65 antibody, and p65 antibody. Results are represented as mean ± SEM, n = 3, * *p* < 0.05, ** *p* < 0.01, *** *p* < 0.001.

To elucidate the effects of DHL on the activation of p38 MAPK and NF-κB induced by LTA in macrophages, RAW264.7 cells (Figure 1A,C–E,I) and primary BMDMs (Figure 1F–H) were used in this study. We first found that DHL did not cause cytotoxicity at concentrations up to 10 μM with 24 h incubation in RAW264.7 cells and BMDMs (Figure 1A,B). Then, cells were treated with DHL (0, 0.3, 1, 3, and 10 μM) for 0.5 h, followed by exposure to LTA for another 0.5 h. Cell lysates were collected, and the phosphorylation of p38 MAPK and NF-κB was detected by Western blot analysis. As displayed in Figure 1C–H, LTA-induced phosphorylation of p38 MAPK and NF-κB was inhibited by DHL in a dose-dependent manner both in RAW264.7 cells and BMDMs. These results demonstrated that DHL inhibits the activation of p38 MAPK and NF-κB in macrophages. Moreover, to identify the effect of DHL on the NF-κB pathway, the degradation of IκBα was detected, and the effect of DHL on the modulation of p-p65 nuclear translocation in LTA-induced inflammation in RAW264.7 cells was also analyzed. The results revealed that DHL inhibits IκBα degradation (Figure 1I) and phosphorylation and nuclear translocation of NF-κB/ p65 (Figure 1J).

### 2.2. DHL Promotes the Activation of AMPK/Nrf2 Pathway in LTA-Induced Macrophages

**Figure 2:**
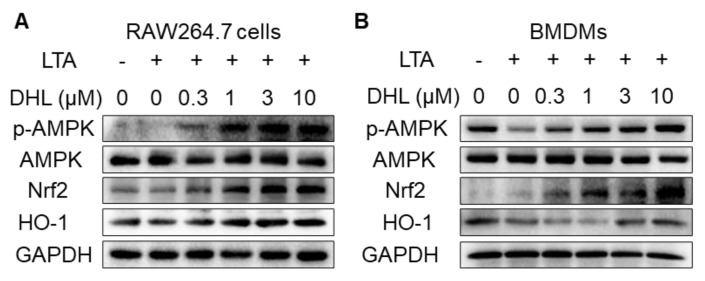

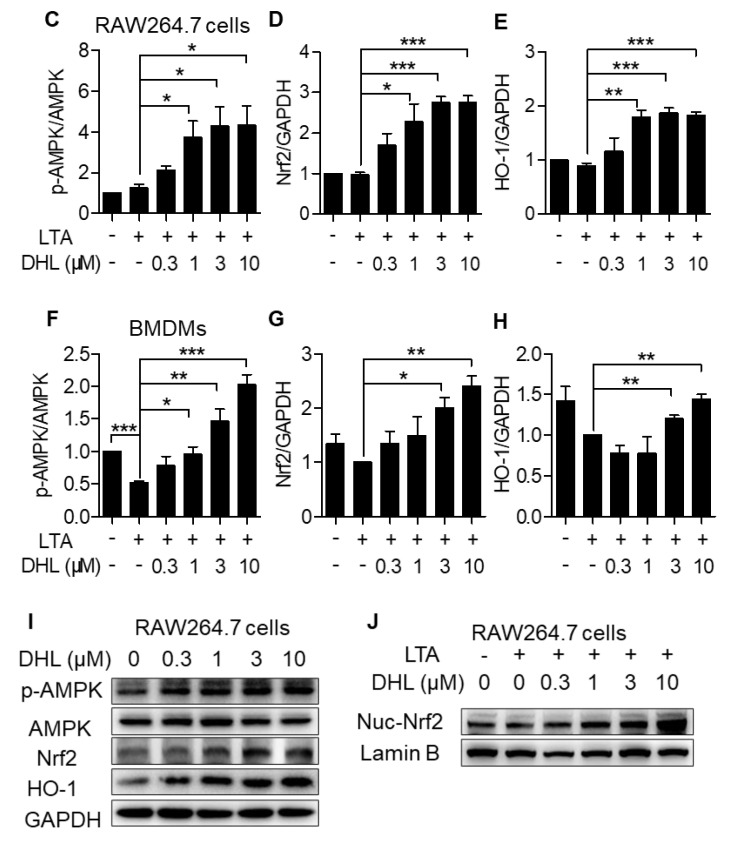
The AMPK/Nrf2 pathway is involved in DHL–mediated macrophage activation by LTA stimuli. RAW264.7 cells (**A**,**C**–**E**) and BMDMs (**B**,**F**–**H**) were preincubated with the indicated concentration (0, 0.3, 1, 3, and 10 μM) of DHL for 0.5 h, followed by LTA (20 μg/mL) stimulation for 0.5 h. (**A**,**E**) Cell lysates were prepared for Western blot analysis and the expression of p-AMPK, AMPK, Nrf2, and HO-1 were detected, with GAPDH used as the loading control. The ratios of p-AMPK/AMPK (**C**,**F**), Nrf2/GAPDH (**D**,**G**) and HO-1/GAPDH (**E**,**H**) were analyzed by ImageJ software. (**I**) RAW264.7 cells were treated with different concentrations (0, 0.3, 1, 3, and 10 μM) of DHL for 0.5 h. Then, cell lysates were prepared to detect the expression of p-AMPK, AMPK, Nrf2, and HO-1, with GAPDH used as the control. (**J**) RAW264.7 cells were pretreated with DHL (0, 0.3, 1, 3, and 10 μM) for 0.5 h, followed by LTA stimulation for another 0.5 h. Nuclear fractions were extracted and subjected to immunoblot analysis using Nrf2 and Lamin B antibodies. Data are represented as mean ± SEM, n = 3, * *p* < 0.05, ** *p* < 0.01, *** *p* < 0.001.

Since Nrf2 signaling plays an important role in macrophage activation, we evaluated the effects of DHL on the Nrf2 signaling pathway in LTA–induced RAW264.7 cells and BMDMs. The phosphorylation of AMPK and the protein content of Nrf2 and HO-1 were detected in this study. As shown in Figure 2B, the phosphorylation of AMPK and the protein levels of Nrf2 and HO-1 were reduced with LTA stimuli in BMDMs. However, DHL promoted the phosphorylation of AMPK and increased the protein levels and gene expressions of Nrf2 and HO-1 in both RAW264.7 cells (Figure 2A,C–E) and BMDMs (Figure 2B,F–H). Additionally, we found that DHL affected the basal levels of the phosphorylation of AMPK and the contents of Nrf2 and HO-1 without LTA stimuli in RAW264.7 cells (Figure 2I). To further investigate whether DHL activated Nrf2 translocation in LTA-induced inflammation, nuclear extracts of RAW264.7 cells were isolated, and the results showed that DHL significantly promoted Nrf2 migration to the nucleus (Figure 2J). These data suggested that the AMPK/Nrf2 signaling pathway is involved in the regulation of DHL on macrophage activation induced by LTA.

### 2.3. DHL Suppresses M1 Phenotype Macrophage Activation While Accelerating M2 Macrophage Polarization in Macrophages

**Figure 3:**
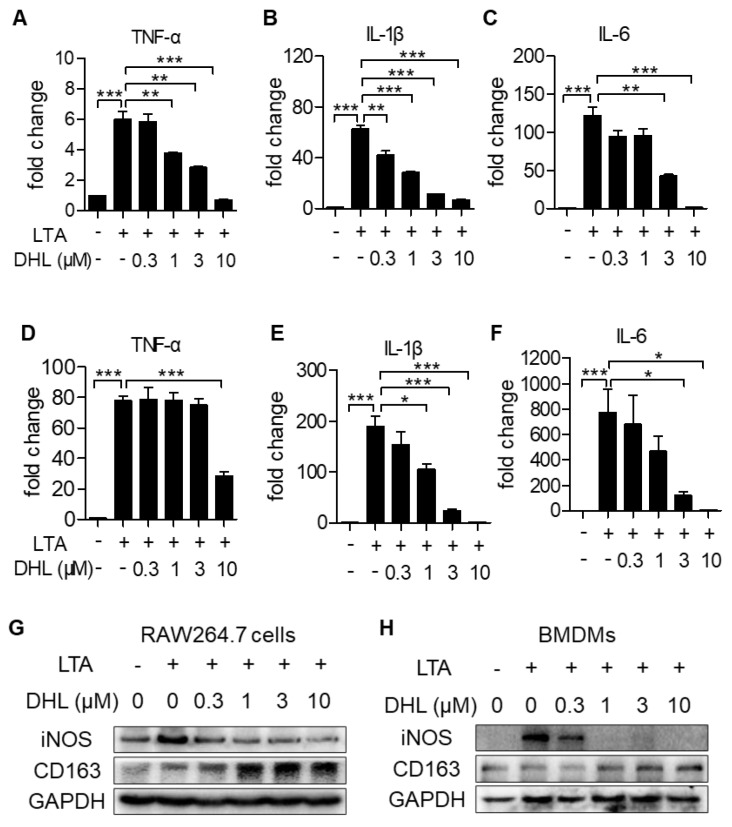

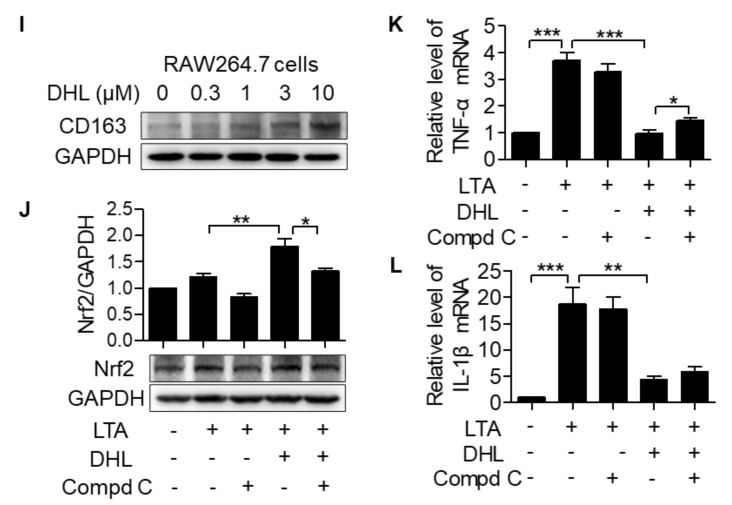
DHL restrains M1 phenotype macrophage activation while accelerating M2 macrophage polarization in RAW 264.7 cells and primary BMDMs. RAW264.7 cells (**A**–**C**) and BMDMs (**D**–**F**) were preincubated with different concentrations of DHL for 0.5 h, followed by stimulation with LTA (20 μg/mL) for 4 h. Cells were collected and total RNAs were isolated, then mRNA levels of M1-like inflammatory cytokines (*TNF-α*, *IL-1β,* and *IL-6*) were measured by real-time PCR. (**G**–**I**) Protein contents of M1 (iNOS) and M2 (CD163) marker genes. RAW264.7 cells (**G**) and BMDMs (**H**) were pretreated with the indicated concentration for 0.5 h, then stimulated with LTA (20 μg/mL) for another 24 h. (**I**) RAW264.7 cells were pretreated with the indicated concentration of DHL for 24 h. Cell lysates were prepared for Western blot analysis, GAPDH was used as loading control. (**J**–**L**) RAW264.7 cells were pretreated with Compound C (Compd C) for 0.5 h, followed by incubation with DHL for 0.5 h, then stimulation with LTA (20 μg/mL) for another 0.5 h (**J**) or 4 h (**K**–**L**). (**J**) Cell lysates were collected for immunoblotting to evaluate the expression of Nrf2, and the ratio of Nrf2/GAPDH was calculated by densitometry. (**K**–**L**) Cells were collected and total RNAs were isolated, then mRNA levels of M1-like inflammatory cytokines (*TNF-α* and *IL-1β*) were measured by real-time PCR. Data are represented as mean ± SEM, n = 3, * *p* < 0.05, ** *p* < 0.01, *** *p* < 0.001.

To explore the effect of DHL on macrophage polarization, the expression of M1 phenotype marker genes (proinflammatory cytokine genes *TNF-α*, *IL-1β,* and *IL-6*) were evaluated. As shown in Figure 3A–F, the mRNA levels of these M1-like inflammatory cytokines triggered by LTA were significantly restrained after treatment with DHL in both RAW264.7 cells (Figure 3A–C) and BMDMs (Figure 3D–F). Moreover, we found that DHL inhibited the protein expression of iNOS (M1 marker gene), but promoted CD163 (M2 marker gene) expression (Figure 3G–I). In addition, we observed that Compd C, an inhibitor of AMPK, could restrain the augmentation of Nrf2 induced by DHL (Figure 3J), and partly reverse the effect of DHL on inhibition of TNF-α and IL-1β (Figure 3K–L). Taken together, these results verified that DHL can modulate the M1/M2 macrophage phenotype, which may partly occur through Nrf2 signaling.

### 2.4. DHL Ameliorates MRSA-Induced ALI in Mice

**Figure 4:**
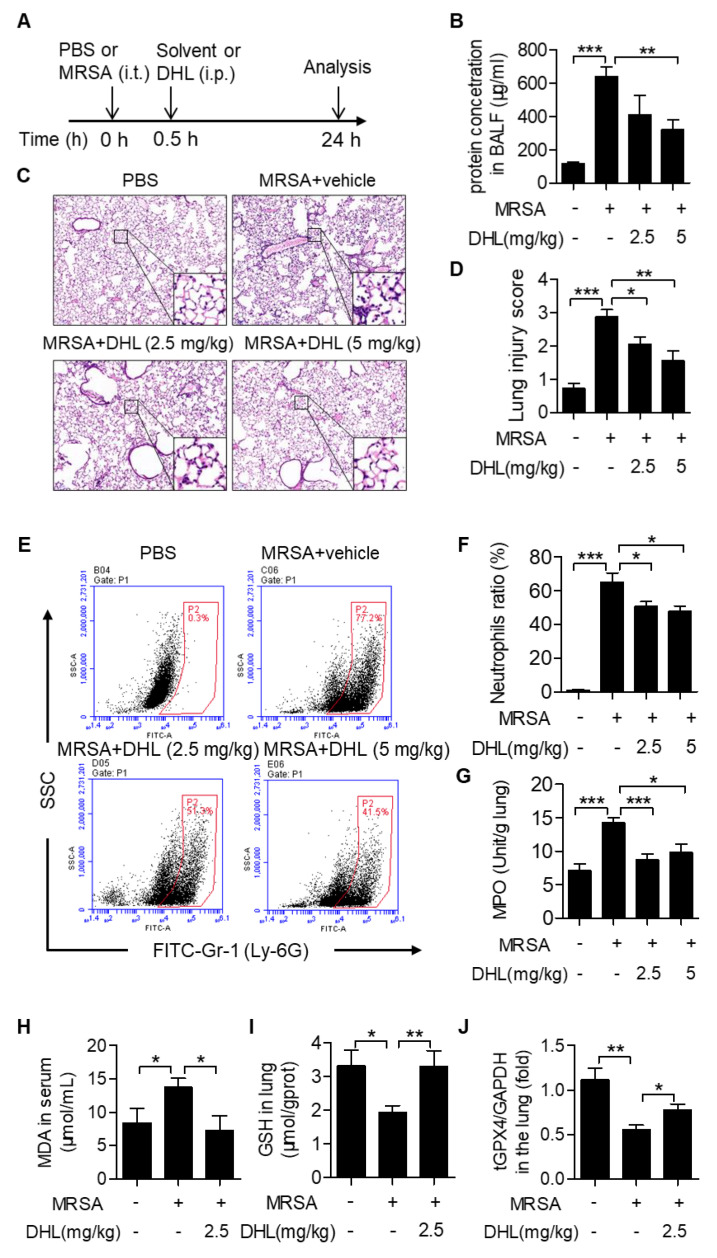
DHL alleviates MRSA-induced ALI in mice. (**A**) The schematic timeline of the MRSA-induced ALI mouse model. C57BL/6 mice were subjected to an intratracheal injection of MRSA (4 × 10^7^ CFU/mouse) for 0.5 h, followed by intraperitoneal injection of vehicle (polyoxyethylene castor oil: ethanol: PBS = 1:1:8) or DHL (2.5 or 5 mg/kg). After MRSA administrated for 24 h, mice were euthanized, and the lung lobes, bronchoalveolar lavage fluid (BALF) and serum were collected. (**B**) The total protein concentration of BALF was measured by a BCA kit. (**C**) Hematoxylin and eosin staining was performed to evaluate the lung histopathological changes (original magnification, 100×). (**D**) Lung tissue injury was evaluated by histological scores in different groups. (**E**) Flow cytometry analysis of the percentage of neutrophil (Ly-6G^+^) in BALF was performed by staining with Ly-6G antibody. (**F**) The statistical analysis of flow cytometry data. (**G**) Myeloperoxidase (MPO) activity in lung tissues was detected. (**H**–**I**) The levels of MDA in serum and GSH in lung tissues were measured by the appropriate detection kit (MDA and GSH assay kit, respectively). (**J**) mRNA expression of tGPX4 in lung tissues was detected by real-time PCR. n = 5, * *p* < 0.05, ** *p* < 0.01, *** *p* < 0.001.

To determine the effects of DHL on Gram-positive bacterial-induced ALI, we established a mouse ALI model by intratracheal injection of MRSA (4 × 10^7^ CFU/mouse). DHL (2.5 and 5 mg/kg) was administered by intraperitoneal injection 0.5 h after MRSA challenge (Figure 4A). The results showed that the BALF protein concentration, an index of lung edema, was prominently increased in MRSA-challenged mice, but decreased in DHL-treated mice (Figure 4B). Histopathological analysis revealed that DHL (2.5 and 5 mg/kg) alleviated the lung lesions in MRSA-injected mice, with decreased inflammatory cell accumulation and alveolar structure destruction (Figure 4C–D). Moreover, infiltration of neutrophils in BAL fluid was determined by staining with anti-Ly6G-FITC (Figure 4E). As shown in Figure 4E–F, DHL administration reduced neutrophil (Ly6G^+^) infiltration induced by MRSA (2.5 and 5 mg/kg). We also detected MPO activity in this study, since it was considered as a specific marker of neutrophils. The results manifested that DHL inhibited MPO activity (Figure 4G), which confirmed that DHL relieved MRSA-induced neutrophil infiltration. However, these results also displayed that the protective effect of a 5 mg/kg dose of DHL on MRSA-induced ALI was not significantly better than that of a 2.5 mg/kg dose, so we chose a 2.5 mg/kg dose of DHL for subsequent analysis. To further identify the effects of DHL on MRSA-induced ALI, the MDA augmentation, GSH, and tGPX4 reduction, which contribute to lung damage, were also assessed. As displayed in Figure 4H–J, DHL inhibited the augmentation of MDA and reversed the decreases in GSH and tGPX4 induced by MRSA in vivo. Taken together, these results demonstrated that DHL significantly alleviates MRSA-induced ALI.

### 2.5. DHL Modulates Macrophage Polarization in MRSA-Induced ALI in Mice

**Figure 5:**
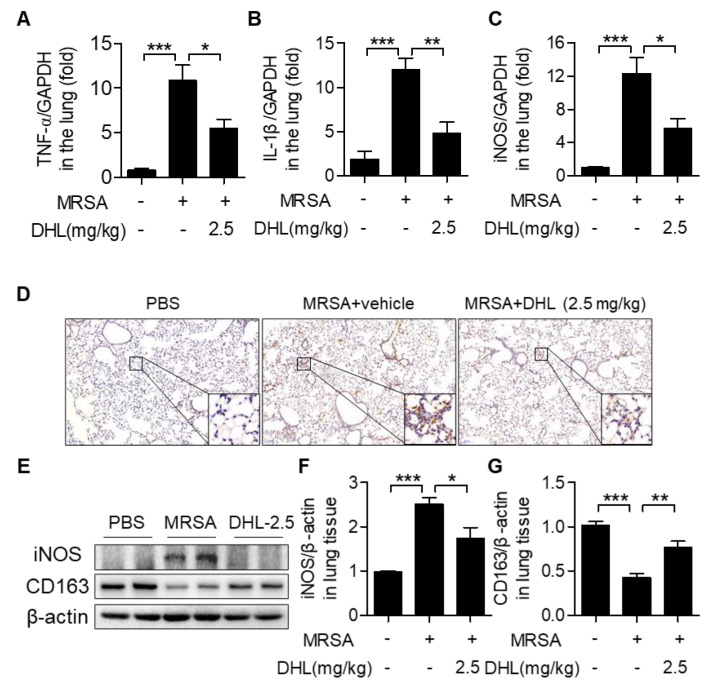

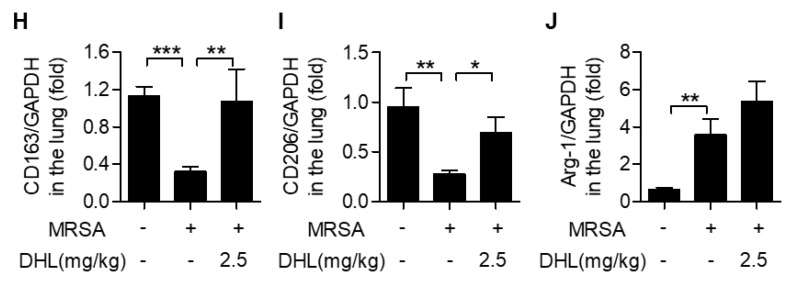
DHL suppresses the MRSA-induced M1/M2 macrophage ratio in mice. Mice were subjected to an intratracheal injection of MRSA (4 × 10^7^ CFU/mouse), and subsequent intraperitoneal injection of vehicle or DHL (2.5 mg/kg) for 24 h. (**A**–**C**) The mRNA levels of M1 phenotype marker genes (TNF-α, IL-1β, and iNOS) in lung tissues were measured by real-time PCR. (**D**) The expression of iNOS in lung tissues was measured by immunohistochemistry (original magnification, 100×). (**E**) The protein expression of M1 phenotype marker gene iNOS and M2 phenotype marker gene CD163 were detected by Western blot; β-actin was used as the loading control. (**F**,**G**) The ratios of iNOS/β-actin (**F**) and CD163/β-actin (**G**) were analyzed. (**H**–**J**) The mRNA levels of M2 marker genes (CD163, CD206 and Arg-1) in lung tissues. n = 5, * *p* < 0.05, ** *p* < 0.01, *** *p* < 0.001.

To evaluate whether DHL protects against MRSA-induced ALI by modulating macrophage polarization, the expressions of M1 and M2 phenotype marker genes in lung tissues were measured. As shown in Figure 5, MRSA injection increased proinflammatory M1 marker gene (TNF-α, IL-1β, and iNOS) expression and downregulated anti-inflammatory M2 marker gene (CD163, CD206, and Arg-1) expression. Conversely, DHL controlled MRSA-induced M1 marker gene (TNF-α, IL-1β, and iNOS) expression, but enhanced M2 marker gene (CD163, CD206, and Arg-1) expression in the lungs of MRSA-injected mice. These data demonstrated that DHL inhibits M1 macrophage polarization but accelerates M2 macrophage polarization in vivo.

### 2.6. DHL Inhibits the Phosphorylation of p38 MAPK and NF-κB, but Promotes the Activation of the AMPK/Nrf2 Pathway in an MRSA-Induced ALI Model

**Figure 6:**
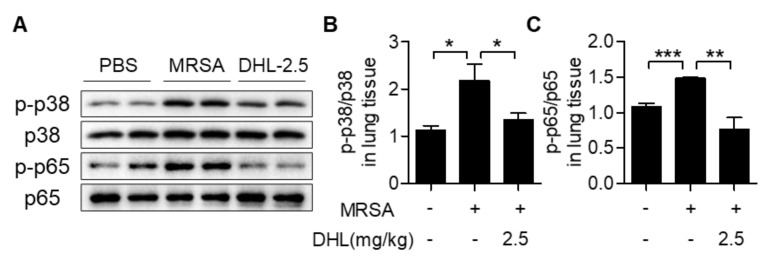

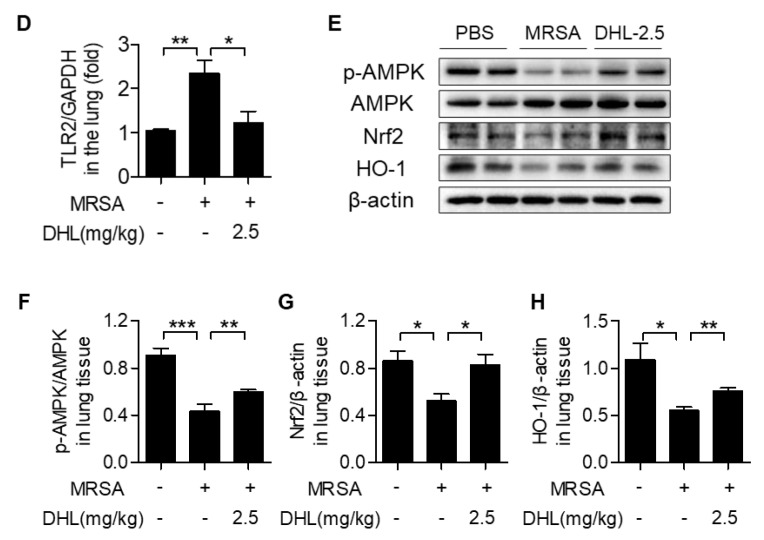
DHL inhibits the TLR2-mediated phosphorylation of p38 MAPK and NF-κB, but promotes the activation of the AMPK/Nrf2 pathway in an MRSA-induced ALI model. Mice were subjected to an intratracheal injection of MRSA (4 × 10^7^ CFU/mouse), and subsequent intraperitoneal injection of vehicle or DHL (2.5 mg/kg) for 24 h. The lung tissues were collected for further analysis. (**A**) The phosphorylation of p38 MAPK and p65 of mouse lung tissues was measured by Western blot; the ratios of p-p38/p38 (**B**) and p-p65/p65 (**C**) were semiquantitatively analyzed using ImageJ software. (**D**) The mRNA level of TLR2 in mouse lung tissues was tested by real-time PCR. (**E**) The phosphorylation of AMPK and the total protein contents of Nrf2 and HO-1 in mouse lung tissues were detected by Western blot; β-actin was used as the loading control. The ratios of p-AMPK/AMPK (**F**), Nrf2/β-actin (**G**), and HO-1/β-actin (**H**) were analyzed by ImageJ software. n = 5, * *p* < 0.05, ** *p* < 0.01, *** *p* < 0.001.

Since the p38 MAPK and NF-κB signals play important roles in M1 macrophage polarization, we detected the activation of p38 MAPK and NF-κB in the lung tissues of MRSA-injected mice. The results verified that the phosphorylation of p38 MAPK and NF-κB triggered by MRSA was significantly inhibited after DHL administration (Figure 6A–C). The expression of TLR2, a receptor of Gram-positive bacteria, which could mediate p38 MAPK and NF-κB activation, was also measured in this study. As shown in Figure 6D, the mRNA expression of TLR2 was induced by MRSA, whereas DHL attenuated the level of TLR2 in lung tissues of mice that had been induced by MRSA. Additionally, we assessed the activation of AMPK/Nrf2 signaling, since it plays a crucial role in macrophage polarization. The results demonstrated that MRSA challenge reduced the phosphorylation of AMPK, and decreased the expression of Nrf2 and its downstream gene HO-1. However, these alternations were reversed by treatment with DHL (Figure 6D–H). Altogether, these results indicated that DHL modulates macrophage activation via inhibiting p38 MAPK and NF-κB phosphorylation, and activating AMPK/Nrf2 signaling.

## 3. Discussion

**Figure 7:**
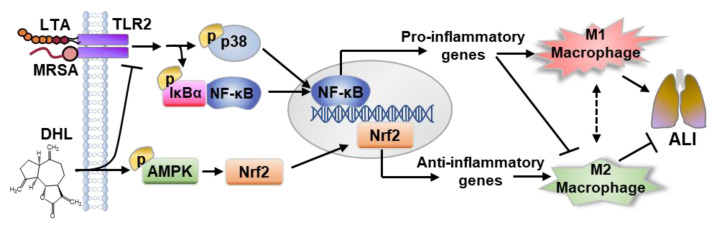
Scheme for the proposed mechanism of DHL in the prevention of MRSA-induced ALI. The mechanism involves DHL inhibiting M1 polarization and promoting M2 polarization, and includes the roles of p38/NF-κB inhibition and AMPK/Nrf2 signaling pathway activation.

This study was designed to investigate the effects of DHL on Gram-positive bacteria-induced macrophage activation and ALI. As depicted in Figure 7, our results demonstrated that DHL inhibited classical M1 polarization, but promoted the alternative M2 polarization of macrophages to suppress Gram-positive bacteria-induced inflammation in vitro and in vivo. Inhibition of p38 MAPK and NF-κB pathways, as well as activation of the AMPK/Nrf2 pathway, played a crucial role in the effect of DHL on promoting M2 macrophage polarization and restraining M1 macrophage polarization, inflammation, and MRSA-induced ALI.

A growing body of evidence suggests that macrophage-mediated inflammation plays a critical role in numerous inflammatory diseases, such as sepsis, ALI, asthma, and pulmonary fibrosis [19,28,29]. Macrophage polarization homeostasis was shown to play an important role in macrophage-mediated inflammation [30]. M1-polarized macrophages can secrete proinflammatory mediators, including TNF-α, IL-1β, and IL-6, and express iNOS; while M2-polarized macrophages express CD163, CD206, and Arg-1, and exhibit anti-inflammatory effects. It was reported that some natural compounds from Chinese herbs exert anti-inflammatory effects via reducing the M1 macrophage ratio and increasing the M2 macrophage ratio [31,32]. DHL is also a natural compound isolated from herbs used in Chinese herbal medicine and possesses a variety of activities including antioxidant, anticancer and anti-inflammatory properties [18,19,22,23]. Previous studies revealed that DHL exerts anti-inflammatory effects and protects against BLM-induced inflammation or LPS (Gram-negative bacterium)-induced ALI via inhibiting M1 macrophage polarization [18,19,24]. However, the effects of DHL on M2 macrophage polarization or Gram-positive bacteria-induced inflammation were not previously well understood. LTA, a major outer wall component of Gram-positive bacteria (e.g., MRSA), has been widely used in studies to seek potential anti-inflammatory candidates [27,33]. In this study, we considered LTA and MRSA as the Gram-positive bacteria for our in vitro and in vivo studies, respectively. We first demonstrated that DHL reduced LTA-induced macrophage inflammation and attenuated MRSA-induced ALI via blocking M1 macrophage polarization as well as promoting M2 macrophage polarization.

It has been verified that Gram-positive bacteria (LTA and *S. aureus*) can trigger an inflammatory response via the TLR2-mediated p38 MAPK and NF-κB pathways [34,35]. When activated, the TLR2/p38 MAPK/NF-κB pathway promotes M1 macrophage polarization and aggravates inflammation; suppression of TLR2-mediated p38 MAPK/NF-κB pathways can decrease M1 macrophage activation and inflammation [36,37,38]. TLR2-deficient mice showed decreased inflammation, and administration of anti-TLR2 monoclonal antibody decreased proinflammatory cytokines’ secretion and increased survival rate of mice suffering from inflammatory response [39,40]. Thus, targeting TLR2 is an effective strategy to improve inflammation. Previous studies manifested that DHL inhibits the TLR4-mediated p38 MAPK and NF-κB signaling pathways, and restrains M1 macrophage polarization as well as the inflammatory response induced by LPS [18]. In this study, we supplemented the knowledge that DHL possesses the abilities to block the Gram-positive bacteria-mediated p38 MAPK/NF-κB pathway and to promote macrophages to switch from the M1 phenotype to M2 phenotype in RAW264.7 macrophages and primary BMDMs in vitro, as well as in a mouse MRSA-ALI model. Furthermore, the mRNA level of TLR2 was reduced after DHL treatment in the MRSA-ALI mouse model, suggesting that DHL might be a therapeutic candidate for Gram-positive bacteria-mediated inflammatory diseases.

AMPK and Nrf2 were also reported to be involved in Gram-positive bacteria-mediated inflammation [41], and activation of the AMPK/Nrf2 signaling pathway could reduce inflammation by enhancing M2 polarization [13]. A previous study found that DHL exhibits the ability to activate Nrf2 [42]; our results make this finding more credible, as DHL promotes the phosphorylation of AMPK, an upstream gene of Nrf2, as well as accelerates the expression and nuclear translocation of Nrf2. HO-1, a downstream gene of Nrf2, has also been demonstrated to alleviate inflammation by altering the balance of M1/M2 macrophages [14,43]. Our data showed that DHL accelerates HO-1 expression in vitro and in vivo. Since the AMPK/Nrf2 signaling pathway plays a crucial role in inflammation, and components isolated from traditional Chinese medicine can attenuate inflammation via activating AMPK/Nrf2 signaling, we believe that DHL might exert anti-inflammation effects partly through AMPK/Nrf2 signaling. Compd C, an AMPK inhibitor, was applied in this study, and verified the speculation described above, as Compd C inhibited the expression of Nrf2 induced by DHL, and reversed the expressions of proinflammatory cytokines (TNF-α and IL-1β) that were suppressed by DHL. MDA, the product of lipid peroxides, is an important indicator of the degree of tissue cell membrane peroxidation [44,45]. GSH is a crucial antioxidant and substrate for GPX4 to protect lipids from peroxidation [46]. Studies revealed that the augmentation of MDA and the reduction of GSH and tGPX4 contribute to lung damage and ALI [47,48]. Moreover, Nrf2 can exert a protective effect in ALI via reducing of MDA and increasing the contents of GSH and tGPX4 [49,50]. In our previous studies, we also found that MRSA increases the contents of MDA [45,51], and that 4-OI, an Nrf2 activator, reversed this phenomenon [45]. The results displayed that DHL inhibited the contents of MDA in the serum, and increased GSH and tGPX4 levels in lung tissues, which confirmed the hypothesis that DHL activates AMPK/Nrf2 signaling and protects against MRSA-induced lung damage.

Interestingly, we found that under stimulation with LTA, the phosphorylation of AMPK decreased in primary BMDMs, but not in RAW264.7 cells. Consistent with primary BMDMs stimulated with LTA, an MRSA challenge resulted in reduced AMPK phosphorylation in lung tissues. Hoogendijk et al. [52] also found that LTA has no significant effect on AMPK phosphorylation in a macrophage cell line. Our previous study showed that the alteration of AMPK phosphorylation was not consistent in RAW264.7 cells and BMDMs upon LPS stimulation [53]. In addition, the phosphorylation of AMPK was not induced in LPS-induced ALI [31], but decreased in D-GalN/LPS-induced acute liver failure [54]. Regardless of the effects of different stimuli on the phosphorylation of AMPK in macrophages and tissues, AMPK agonists are able to improve the damage to macrophages and tissues under these stimuli [31,52,53,54], indicating that activation of AMPK might be a vital index for seeking potential candidates with anti-inflammatory activities and improving tissue damage.

Chinese herbal medicine has been widely used in treating diseases for centuries. DHL, the main active ingredient of *Saussurea lappa* and *Inula helenium* L., has been reported to inhibit the secretion of proinflammatory cytokines (e.g., TNF-α, IL-1β, and IL-6) and alleviate LPS-induced ALI by modulating macrophage activation [18,24]. However, whether DHL influences Gram-positive bacteria-induced inflammation and ALI is still unknown. *S aureus* is a notorious Gram-positive bacterium that causes a variety of inflammatory diseases. Concerningly, most *S. aureus* are already resistant to all antibiotics; thus, we selected methicillin-resistant *S. aureus* (MRSA) for our in vivo study to find potential drug candidates for treating methicillin-resistant Gram-positive bacterial infection. During infection, neutrophils are the earliest immune cells to be recruited to the injury site, resulting in alveoli basement membrane destruction, edema formation, and proinflammatory cytokine production, eventually causing lung damage [55]. Thus, neutrophil infiltration is a critical marker of acute pulmonary inflammation; inhibition of neutrophil infiltration can effectively ameliorate inflammation and lung injury. For instance, Zhu et al. [56] found that partial neutrophil depletion led to declined pulmonary inflammation. In this study, we proved that DHL inhibits neutrophil accumulation, decreases proinflammatory mediators’ production, and alleviates MRSA-induced ALI. Moreover, DHL was found to exert these effects via regulating macrophage polarization through the p38 MAPK/NF-κB and AMPK/Nrf2 signaling pathways. The p38 MAPK/NF-κB and AMPK/Nrf2 signaling pathways are also related to endothelial and epithelial activation [57,58,59,60], and may contribute to enhancing neutrophil infiltration and pulmonary damage [61,62]. Therefore, further studies should focus on whether DHL provides protective effects on endothelial and epithelial activation.

In conclusion, our study, for the first time, demonstrated that DHL significantly reduced M1 polarization and enhanced M2 polarization in Gram-positive bacteria-challenged macrophages through inhibiting the p38 MAPK/NF-κB pathway and activating AMPK/Nrf2 signaling. Moreover, DHL ameliorated MRSA-induced ALI via promoting macrophage M2 polarization. Taken together, our results suggested that DHL might be a potential anti-inflammatory agent beneficial for treating Gram-positive bacteria-induced ALI/ARDS and sepsis patients.

## 4. Materials and Methods

### 4.1. Reagents and Antibodies

Dehydrocostus lactone (C_15_H_18_O_2_; MW: 230.30; purity ≥ 99%) (Figure 1A) and Compound C (Compd C) were purchased from Target Molecule Corp. (Target Mol, Shanghai, China). A stock solution of DHL (for in vitro study) was prepared at a concentration of 10 mmol/L in dimethyl sulfoxide (DMSO) (Sigma, St Louis, MO, USA) and stored at −20 °C. Lipoteichoic acid (LTA) from *Staphylococcus aureus* (L2515) was obtained from Sigma-Aldrich (St. Louis, MO, USA). Primary antibodies against p-p65, p65, p-p38 MAPK, p38 MAPK, p-AMPK(Thr172), AMPK, Nrf2, IκBα, and GAPDH were purchased from Cell Signaling Technology (Danvers, MA, USA). HO-1, iNOS, and CD163 were acquired from Abcam (Cambridge, MA, USA). β-actin and Lamin B were obtained from Santa Cruz Biotechnology (Dallas, TX, USA). CD16/32 antibody and FITC-conjugated anti-Ly-6G antibody were purchased from BD Biosciences (San Jose, CA, USA).

### 4.2. Cell Culture and Treatment

Bone-marrow-derived macrophages (BMDMs) were isolated from C57BL/6 mice, and mice femurs and tibias were flushed with PBS and cultured in high-glucose DMEM medium (Gibco-BRL, Grand Island, NY, USA) supplemented with 10% fetal bovine serum (Gibco-BRL, Grand Island, NY, USA), 1% penicillin–streptomycin (Gibco-BRL, Grand Island, NY, USA) and macrophage colony-stimulating factor (M-CSF, 10 ng/mL) (PeproTech, Rocky Hill, NJ, USA) for 6 days in an incubator (37 °C, 5% CO_2_) as we previously described [53]. Then, cells were harvested and pretreated with DHL for 0.5 h before stimulation with LTA (20 μg/mL) [45] for the indicated time. Murine macrophage cell line RAW264.7 cells (ATCC, Manassas, VA, USA) were cultured in RPMI-1640 media containing 10% FBS (Gibco-BRL, Grand Island, NY, USA) and 1% penicillin–streptomycin (Gibco-BRL, Grand Island, NY, USA). Cells were incubated in an incubator (37 °C, 5% CO_2_) and treated with indicated concentrations of DHL according to experiment requirements; 0.1% DMSO was added to the culture media as the solvent control. Cells were collected for mRNA and protein analysis.

### 4.3. Cell Viability Assay

Cells were seeded into 96-well plates (1 × 10^4^ cells/well) in the presence or absence of DHL (0–10 μM) for 24 h. Cell viability was tested with an CCK-8 Cell Counting Kit (Biosharp, Hefei) according to the manufacturer’s instructions. The absorbance (Abs.) was detected at 450 nm on a microplate reader (Synergy H4, BioTek, VT, USA). Cell viability was calculated using the following equation: Cell viability (%) = (Abs. of treatment group/Abs. of control group) × 100%.

### 4.4. Preparation of MRSA

The MRSA (ATCC43300) strain was cultured and harvested according to our previously reported methods [36]. Briefly, a single colony of MRSA was picked and transferred into liquid Bertani (LB) medium and cultured with shaking (200 rpm/min) overnight at 37 °C. Subsequently, the bacterial suspension was diluted 100-fold in liquid LB medium and grown with shaking (200 rpm/min) at 37 °C until the bacteria suspension reached the mid-log phase (O.D. 600 nm). Then the suspension was centrifuged and washed with PBS, and the pellet was resuspended by sterile PBS. The MRSA concentration was determined by serially diluting the suspension on LB-agar followed by counting colonies on LB-agar.

### 4.5. Animals

Male C57BL/6 mice (8–10 weeks of age, specific pathogen free) were purchased from Slac Laboratory Animal Co. Ltd. (Shanghai, China). The animals were housed at five per cage in pathogen-free conditions in a climate-controlled room with a 12 h light/dark cycle. All the animal experimental procedures in this study were approved by the Instructional Animal Care and Use Committee (IACUC) of Jiangnan University (JN.No20191230c04009 01, approval date 15 January 2020).

### 4.6. MRSA-Induced ALI Mouse Model

Male C57BL/6 mice were randomly divided into four groups: PBS group, MRSA+vehicle group, MRSA+DHL (2.5 mg/kg) group, and MRSA+DHL (5 mg/kg) group [18,19]. After anesthesia with pentobarbital sodium, mice were intratracheally (i.t.) injected with PBS or MRSA (4 × 10^7^ CFU/mouse) in 50 μL PBS. For MRSA+DHL groups, DHL (2.5 or 5 mg/kg) was intraperitoneally (i.p.) injected 0.5 h after MRSA challenge. All mice were euthanized 24 h after MRSA exposure; serum, bronchoalveolar lavage, and lung tissues were collected immediately for subsequent studies.

### 4.7. Lung Histological Assay

Lung tissues were fixed in 4% paraformaldehyde for 48 h, embedded in paraffin, and cut into 4 μm sections. The slides were stained with hematoxylin and eosin (H&E). Subsequently, sections were assessed with a Pannoramic MIDI (3D, HISTECH, Budapest, Hungary) as we described previously [53]. Ten fields from lung sections were randomly selected to determine lung injury degree, and each field was scored as 0–4 according to the severity of damage as described previously [63]: grade 0: normal appearance, no injury; grade 1: mild polymorphonuclear leukocyte infiltration (PMN) and interstitial congestion; grade 2: moderate cell infiltration, perivascular edema, and moderate destruction of lung structure; grade 3: massive cell infiltration and moderate lung alveolar damage; grade 4: severe cell infiltration and destruction of lung structure.

### 4.8. Flow Cytometry Assay

Bronchoalveolar lavage fluid (BALF) cells were incubated with anti-mouse CD16/32 antibody to block the Fc receptor. The populations of neutrophils (Ly6G^+^) in each BALF sample were stained with FITC-conjugated Ly6G and analyzed by flow cytometer (BD Accuri C6, BD Biosciences, San Jose, CA, USA), then depicted using FlowJo software [36,64].

### 4.9. Myeloperoxidase Activity Assay

Lung tissues were flushed with PBS, then homogenized to measure the MPO activity using an MPO test kit (Nanjing Jiancheng Bioengineering Bio Co., Ltd., Nanjing, China) according to the manufacturer’s instructions.

### 4.10. Measurement of MDA and GSH Contents

The peripheral blood was collected and centrifuged, and then malondialdehyde (MDA) levels in serum were tested using an MDA assay kit (Nanjing Jiancheng Bioengineering Bio Co., Ltd., Nanjing, China). Lung tissues were homogenized, and the whole lysates were harvested to measure the GSH content using Reduced Glutathione Assay Kits (Nanjing Jiancheng Bioengineering Bio Co., Ltd., Nanjing, China) according to the manufacturer’s instructions [53].

### 4.11. Quantitative RT-PCR Analysis

Total RNA of macrophages and lung tissues was isolated using TRIzol Reagent (Life Technologies, Waltham, MA, USA). The cDNA was prepared with a PrimerScript RT Reagent kit (TaKaRa, Otsu, Shiga, Japan). The mRNA expression of target genes was measured by real-time PCR using SYBR Premix Ex Taq TM (TaKaRa, Otsu, Shiga, Japan) and normalized to GAPDH. Quantification was performed using the 2^(−ΔΔCt)^ method. The primer sequences are shown in Table 1.

### 4.12. Immunoblotting

The immunoblotting was performed as described previously [53]. Total protein was extracted from treated cells and lung tissues that were lysed by a RIPA reagent (BioSharp, Hefei, China) containing 1% protease and phosphatase inhibitor cocktail (MedChem Express, Monmouth Junction, NJ, USA). For investigation of nuclear p-p65 and Nrf2 expression, the nuclear fraction was obtained using nuclear extraction regents (Thermo Fisher Scientific, Waltham, MA, USA). Total protein concentration of each sample was measured using a BCA protein assay kit (Beyotime, Shanghai, China). Equal amounts of proteins were resolved on 10% gradient SDS-polyacrylamide gel and transferred to nitrocellulose membranes (Millipore, Billerica, MA, USA). After blocking with 5% nonfat dry milk for 1 h at room temperature, the proteins were incubated with appropriated primary antibodies (1:1000) at 4 °C overnight. Membranes were washed with 1× TBST three times and then incubated with the appropriate HRP-conjugated secondary antibody (1:10,000) at RT for 1 h. Blots were visualized with an ECL kit (Millipore, Billerica, MA, USA) using a ChemiDoc MP Imaging System (Bio-Rad Laboratories, Hercules, CA, USA), and the band densities were quantified by ImageJ software (National Institutes of Health, Bethesda, MD, USA).

### 4.13. Immunohistochemistry

Lung sections were deparaffinized and rehydrated, and immunostaining was performed with a Streptavidin-Biotin Complex immunohistochemical assay kit (Wuhan Boster Technology, Ltd., Wuhan, China). The primary antibody against iNOS (Abcam, Cambridge, MA, USA)) was used in this study at dilution of 1:200. Images were captured using a Pannoramic MIDI (3D HISTECH, Budapest, Hungary) and analyzed with Pannoramic Viewer software (3D HISTECH).

### 4.14. Statistical Analysis

Data are presented as mean ± SEM of at least three independent experiments. Differences between two groups were compared by Student’s t-test, and comparisons among multiple groups were carried out by one-way ANOVA analysis (Tukey’s post hoc test). Statistical analyses were performed using Prism 7 (GraphPad, San Diego, CA, USA); *p* < 0.05 was considered as statistically significant.

## Figures and Tables

**Table 1:** Primer sequences of the target genes.

Target Gene	Primer	Primer Sequences (5′–3′)
GAPDH	Forward	TGGCCTTCCGTGTTCCTAC
	Reverse	GAGTTGCTGTTGAAGTCGCA
TNF-α	Forward	CCTGTAGCCCACGTCGTAG
	Reverse	GGGAGTAGACAAGGTACAACCC
IL-1β	Forward	GAAATGCCACCTT TTGACAGTG
	Reverse	TGGATGCTCTCATCAG GACAG
IL-6	Forward	CTGCAAGAGACTTCCATCCAG
	Reverse	AGTGGTATAGACAGGTCTG TTGG
tGPX4	Forward	CGCAGCCGTTCTTATCAATG
	Reverse	CACTGTGGAAATGGATGAAAGTC
iNOS	Forward	GTTCTCAGCCCAACAATACAAGA
	Reverse	GTGGACGGGTCGATGTCAC
CD163	Forward	ATGGGTGGACACAGAATGGTT
	Reverse	CAGGAGCGTTAGTGACAGCAG
CD206	Forward	CTCTGTTCAGCTATTGGACGC
	Reverse	TGGCACTCCCAAACATAATTTGA
Arg-1	Forward	CTCCAAGCCAAAGTCCTTAGAG
	Reverse	GGAGCTGTCATTAGGGACATCA
TLR2	Forward	TCTAAAGTCGATCCGCGACAT
	Reverse	CTACGGGCAGTGGTGAAAACT

## Data Availability

The data presented in this study are available upon request from the corresponding author.

## Abbreviations

ALI	acute lung injury
AMPK	adenosine monophosphate-activated protein kinase
ARDS	acute respiratory distress syndrome
Arg-1	arginase-1
BALF	bronchoalveolar lavage fluid
BMDM	bone-marrow-derived macrophage
GSH	glutathione
H&E	hematoxylin and eosin
HO-1	heme oxygenase-1
IκBα	inhibitor of nuclear factor-κB alpha
IL-1β	interleukin-1 beta
IL-6	interleukin-6
iNOS	inducible nitric oxide synthase
LTA	lipoteichoic acid
M1	classically activated macrophage
M2	alternatively activated macrophage
MAPK	mitogen-activated protein kinase
MDA	malondialdehyde
MPO	myeloperoxidase
MRSA	methicillin-resistant *Staphylococcus aureus*
NF-κB	nuclear factor-κB
Nrf2	nuclear factor erythroid 2-releated factor 2
tGPX4	total glutathione peroxidase 4
TLR2	toll-like receptor 2
TNF-α	tumor necrosis factor-alpha
